# Correction: Optogenetically induced low-frequency correlations impair perception

**DOI:** 10.7554/eLife.55718

**Published:** 2020-07-13

**Authors:** Anirvan S Nandy, Jonathan J Nassi, Monika P Jadi, John H Reynolds

## Application of the Wilcoxon Signed Rank Test

After publication, the authors became aware that the index used to quantify the behavioral effect of laser stimulation (PDMI, perceptual discrimination modulation index) was not normally distributed according to a Lilliefors test of normality (p<0.05). We have therefore reanalyzed the data using the appropriate non-parametric hypothesis test -- the Wilcoxon sign-rank test. The experiment was designed to test the hypothesis that low-frequency correlated variability would impair orientation discrimination (corresponding to a median PDMI > 0). A one-tailed Wilcoxon sign-rank test rejected the null hypothesis that the PDMI is not > 0, supporting the conclusion that low-frequency laser stimulation impaired orientation discrimination. Applying the same test to data collected (a) during high-frequency stimulation or (b) when the monkey performed the task on stimuli appearing away from the opsin site, we find no evidence of impairment, supporting our original conclusion that the impairment is limited to low-frequency stimulation and is spatially specific to the retinotopic location of the opsin site.

## Analysis of psychometric fits using a Weibull function

The p-value from the Wilcoxon sign-rank test (p=0.048) was close to our significance threshold (α=0.05). We therefore undertook additional analyses to test the robustness of these conclusions. First, we repeated our analyses using an alternative psychometric function. We applied the Weibull function in place of the logistic function that was used in our initial analyses. The Weibull function is arguably a better choice than the logistic function. It is more widely used in the psychophysical literature due, in part, to the fact that the threshold parameter (alpha) in the Weibull function is decoupled from the other parameters (slope, guessing rate). In other words, the shape of the Weibull depends only on the slope and the guessing rate. Varying the threshold parameter, alpha, shifts the function along the x-axis without changing its shape. This decoupling therefore enables independent estimates of the threshold and slope (Nachmias, 1981). The logistic function, though similar in shape, does not decouple these parameters.

After recalculating the PDMI values using Weibull fits we find that the PDMI distribution continues to be non-normal (Lilliefors test) so we again applied the Wilcoxon sign-rank test. As with the PDMI values derived from logistic fits, the Weibull fits show a significant increase in the perceptual threshold for orientation discrimination when introducing low-frequency laser stimulation for stimuli appearing at the retinotopic location of the opsin site (Wilcoxon sign-rank test, p=0.01). There was no significant impairment using high-frequency stimulation (p=0.89) and no significant impairment for stimuli appearing at the non-opsin site (p=0.49) during low-frequency stimulation. Thus, these analyses support the main conclusions of the paper — that low-frequency stimulation (but not high-frequency stimulation) impairs perception and that this effect is specific to the opsin site.

## Analysis of PDMI within the framework of Estimation Statistics

In order to provide readers with a deeper understanding of the data, we also reanalyzed the data within the framework of Estimation Statistics (ES). This approach, which has been widely adopted in some scientific disciplines, provides a richer representation of the data than the more commonly used Null-Hypothesis Significance Testing (NHST) approach and is being adopted as a new standard in some neuroscience journals (Bernard, 2019).

As articulated in the work of Calin-Jageman and Cumming (Calin-Jageman and Cumming, 2019), the Estimation Statistics approach is complementary to the NHST approach, and provides a principled way to measure effect sizes coupled with estimates of uncertainty, yielding interval estimates of effect size.

We applied this approach as follows. For each orientation change condition $\Delta_{ori}$, we calculated the hit rate as the ratio of the number of trials on which the monkey correctly identified (made a saccade to) the target (i.e., the number of hits) divided by the number of trials on which the target was presented. We defined a false-alarm as a trial on which the monkey made an eye-movement to a non-target stimulus at the cued location, within the same time window, relative to stimulus onset, that was applied to targets when detecting hits.

There are several possible ways of estimating false alarm (FA) rates in the context of the behavioral task used in this study. To test whether the conclusions of the paper depend on this choice, we carried out the ES analysis for each approach. Estimates of FA rates were computed separately for each session. There was no difference in FA rates across laser and no-laser conditions, so FA rates were averaged across these conditions, below.

1. In the first approach the lower asymptote of the Weibull function was treated as a free parameter, as was done in the published manuscript.
2. In the second approach the lower asymptote was constrained by false alarm rates computed as the fraction of trials on which the monkey made an eye movement to a non-target (i.e., # of false alarms / # of trials).
3. In the third approach the lower asymptote was constrained by false alarm rates computed as the probability of the monkey making a saccade to a non-target for each non-target (i.e., # of false alarms / # of non-targets). This treats each non-target as a perceptual decision that could trigger a false alarm.
4. In the fourth approach the lower asymptote was constrained by false alarm rates computed during catch trials (i.e., # of false alarms on catch trials / # of catch trials).
5. In the fifth approach the lower asymptote was constrained by a guessing rate. The guessing rate was taken as the reciprocal of the average number of stimuli that were presented across trials.

For each of these five approaches, we obtained the maximum likelihood fits to 20 jackknives of the corresponding data (with 5% of trials left out for each jackknife). The threshold value for each experimental condition was taken as the average across the 20 jackknife fits. The results of these five different fitting approaches appear in the Figure below. FA rates were slightly but significantly higher when attention was directed toward the opsin site, so FA rates were computed separately for each attention condition (attend to opsin site [‘attend-in’], attend away from opsin site [‘attend-away’]). Repeating these analyses by computing FA rates from trials collapsed across attention conditions did not materially change the result.

**Figure 1.**
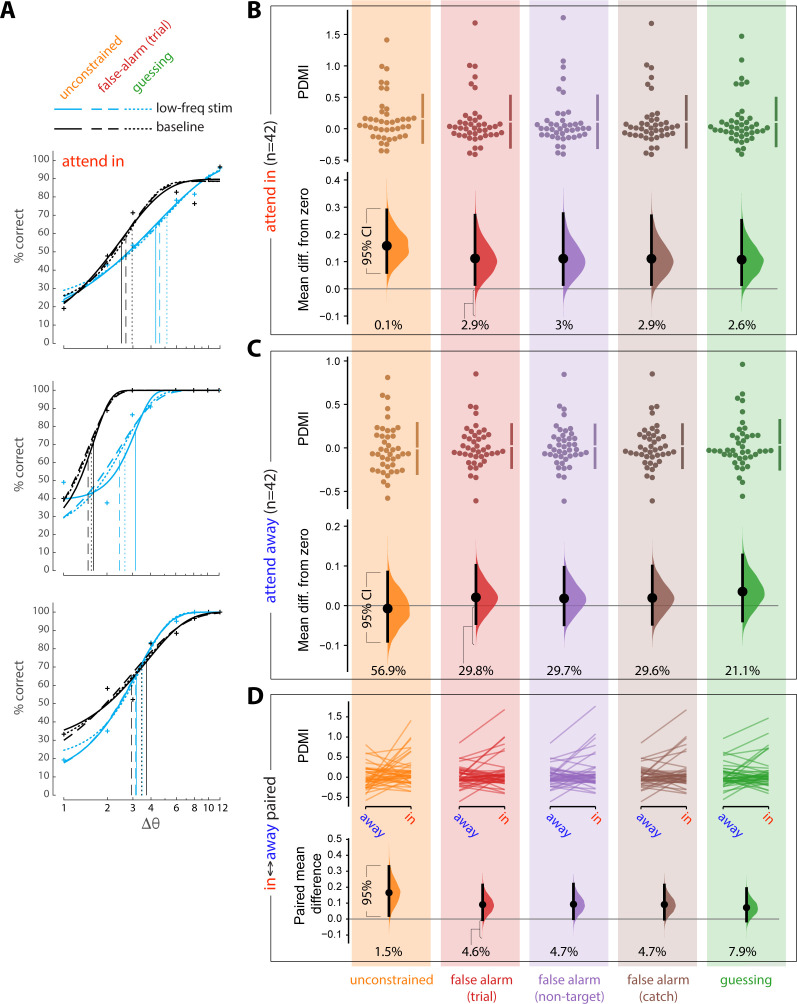


Panel **A** shows fits for three example sessions (the same sessions as the examples in the main text Figure 2B, Figure 2—figure supplement 2B). Blue lines correspond to the low-frequency laser stimulation condition. Black lines correspond to the baseline (no laser stimulation) condition. Solid lines show unconstrained fits (data shown in orange, Panels B-D). Dashed lines show fits constrained to false alarm rates computed by trial (data shown in red, Panels B-D). Dotted lines show fits constrained to guessing rate (data shown in green, Panels B-D). Vertical lines show the corresponding estimates of threshold. Fits for the remaining two estimates of FA rate, not shown, were similar.

Lapse rates (defined to be the fraction of targets that went undetected at the largest orientation change, 12^o^) did not differ between the laser and no-laser conditions, in either attention condition (p=0.687, p=0.69; two-tailed permutation test). To avoid over-constraining the fits and obtaining erroneous estimates of threshold and slope (Wichmann and Hill, 2001), we left the lapse rate as a free parameter during the fitting procedures.

PDMI values were calculated, for each session, from the estimated thresholds separately for the five different fitting regimes.

We next analyzed the PDMI values using the estimation statistics framework (https://www.estimationstats.com) to derive confidence intervals on the magnitude of the effect of low-frequency laser stimulation on perceptual threshold. We evaluated the mean difference from zero of the PDMI values when the monkeys performed the task at the opsin site (‘attend-in’ condition) and when they performed the same task at a non-opsin site (‘attend-away’ condition). These are shown in Panels **B** and **C** respectively. The upper part of each panel shows the raw PDMI values for the five fitting procedures described above. The lower part shows the mean difference from zero of these distributions, plotted as bootstrap sampling distributions (Calin-Jageman and Cumming, 2019; Ho et al., 2019), indicating the distribution of effect size that is compatible with the data. 95% confidence intervals are indicated by the black vertical bars. The value below each distribution indicates the percentage of the distribution that is below zero.

Less than 5% of the effect size distribution fell below zero for the attend-in condition for all fitting methods (Panel **B**), supporting the conclusion that low-frequency laser stimulation causes an impairment in orientation discrimination.

Panel **C** shows the results of the identical analyses applied to stimuli appearing at the retinotopic location of the non-opsin site. The 95% confidence intervals contain zero for the attend away condition for all fitting methods, providing no indication that low-frequency laser stimulation altered orientation discrimination at the non-opsin site.

From these analyses, we conclude that induction of low-frequency correlations at the opsin site impairs orientation discrimination for stimuli appearing at the retinotopic location of the opsin site, and causes no measurable impairment at the non-opsin site. This finding holds across five different approaches to treating FA rates.

To further assess the spatial selectivity of the impairment, we compared PDMI values across the two attention conditions. Panel **D** shows the results of the session-wise paired difference in PDMI values across the two attention conditions. 95% or more of the distribution is above zero for four of the analysis. If we constrain the psychometric function by a FA rate estimated as the guessing rate, however, 92.1% of the effect size distribution falls above zero and 7.9% falls below zero. Thus, while we do find compelling evidence for an impairment at the opsin site, the evidence is mixed as to whether this impairment is entirely limited to that site.

## Timing analysis

In the course of reanalyzing the data, we also examined whether our estimate of false alarm rate could have been influenced by the differential timing of targets and distracters (non-targets). In the task used to estimate orientation discrimination thresholds, target stimuli always occurred after at least one non-target. Since the average time of targets and non-targets differed, a change in the monkey’s strategy over time could differentially affect the likelihood of hits and false alarms. If so, the lower asymptote of the psychometric function, which was fixed by the false alarm rate in some analyses (see red distributions, above figure) could reflect a strategy that was somewhat different from hit rates (the rest of the data used to fit the psychometric function). To examine this, we computed false alarm rates over 500 msec. windows centered on the average onset time of targets (2445 msec. after fixation onset) and non-targets (1531 msec. after fixation onset). False alarm rates did not differ significantly (7.4%, window centered on 2445 msec.; 7.3%, window centered over 1531 msec.; Wilcoxon Signed-Rank test, p=0.64).
